# Prosthetic, orthodontic and implant-supported rehabilitation of five maxillary anterior teeth with alveolar bone loss

**DOI:** 10.1590/2177-6709.23.1.087-096.oar

**Published:** 2018

**Authors:** Odilon Guariza-Filho, Cristiano Miranda de Araujo, Angela Graciela Deliga Schroder, Orlando Motohiro Tanaka, Ricardo Kern, Antonio Carlos Ruellas

**Affiliations:** 1 Pontifícia Universidade Católica, Faculdade de Odontologia, Departamento de Ortodontia (Curitiba/PR, Brazil).; 2 ​Private practice (Curitiba/PR, Brazil).; 3 Universidade Federal do Rio de Janeiro, Faculdade de Odontologia, Departamento de Ortodontia (Rio de Janeiro/RJ, Brazil).

**Keywords:** Dental implants, Interdisciplinary treatment, Orthodontics, Prosthetic, Rehabilitation

## Abstract

**Introduction:**

Treatment of maxillofacial injuries is complex and requires the establishment of a comprehensive and accurate diagnosis and correct treatment planning.

**Objective:**

The objective of this case report was to describe the re-treatment of a 27-year-old woman who was involved in a severe car accident that resulted in the loss of five anterior teeth and alveolar bone, and whose previous orthodontic and surgical treatments had been unsuccessful.

**Case report:**

In this case, the space for the missing mandibular molar was reopened to allow for rehabilitation. The positions of the mandibular incisors were improved. The right mandibular canine was moved to the mesial, allowing for correction of the Class II canine relationship on that side, and implants were placed to replace the maxillary anterior teeth.

**Conclusion:**

Anterior aesthetic and functional rehabilitation using a multidisciplinary approach was essential to improve the patient’s facial aesthetics, to obtain great improvement in function and to achieve occlusal stability after 2 years of follow-up.

## RESUMO

**Introdução:**

o tratamento de danos bucomaxilofaciais é complexo e requer diagnóstico abrangente e preciso, além de um correto plano de tratamento.

**Objetivo:**

o objetivo deste relato de caso foi descrever o retratamento de uma paciente do sexo feminino, 27 anos de idade, envolvida em acidente automobilístico grave, que resultou na perda de cinco dentes, além de perda óssea alveolar na região anterior.

**Relato de caso:**

a paciente apresentava histórico de insucesso de tratamento ortodôntico e cirúrgico. No caso apresentado, o espaço referente aos molares inferiores ausentes foi reaberto, para possibilitar a reabilitação. Houve melhora no posicionamento dos incisivos inferiores. O canino inferior direito foi deslocado para mesial, possibilitando a correção da relação de Classe II intercaninos do mesmo lado. Implantes foram inseridos a fim de substituir os dentes superiores da região anterior.

**Conclusão:**

a reabilitação estética e funcional da região anterior, realizada por meio de uma abordagem multidisciplinar, foi fundamental para valorizar a estética facial da paciente, melhorar a função e promover estabilidade oclusal após dois anos de acompanhamento.

## INTRODUCTION

An interdisciplinary approach is often indicated in the planning and treatment of patients with severe maxillofacial trauma.^1^ Traumatic injuries (e.g., accidents involving motor vehicles, firearms, and falls) frequently result in anterior tooth loss or tooth fractures,^2^ ^,^ ^3^ which subsequently lead to aesthetic, functional, and speech problems.^4^

Therapy begins with an understanding of the patient’s desires. In most cases, the patient’s primary desire is aesthetic tooth replacement to achieve a pleasant smile. For the dental clinician, the reestablishment of aesthetics and function requires knowledge of all treatment modalities. Among the fixed options, conventional fixed partial dentures and implant-supported restorations should be objectively evaluated for their potential to provide long-term function and stability in a given situation. Implant-supported restorations are often the best solution because higid tooth structure and supporting tissues can be preserved.^5^ When there is limited space for implantation or pre-existing malocclusion, orthodontic treatment may be necessary to achieve a good result.^6^ ^,^ ^7^

For successful anterior aesthetic and functional rehabilitation and implant restoration, the following factors must be considered: the interarch space, existing occlusal plane, arches relationship, implant position, arch form, existing occlusion and prosthesis, number and location of missing teeth, lip line, and mandibular flexure.^8^ Following careful clinical and radiographic examinations and establishment of the correct diagnosis, the orthodontist, surgeon, and prosthodontist should collectively establish a treatment plan.^7^

In the present study, the clinical case of an adult who suffered a car accident that resulted in the loss of all maxillary incisors will be reported. The aim of this report was to describe and discuss the treatment of this patient and the 2 years of successful retention, for which a multidisciplinary approach was essential to achieve aesthetic and functional success, with an increase in the patient’s self-esteem. 

## CASE REPORT

The patient was a 27-year-old Caucasian woman with a history of previous orthodontic treatment. A severe car accident led to the loss of five anterior teeth and alveolar bone. The patient had been unsuccessfully treated orthodontically; an alveolar bone graft had been previously placed. 

### Diagnosis and treatment plan

**Figure 1:**
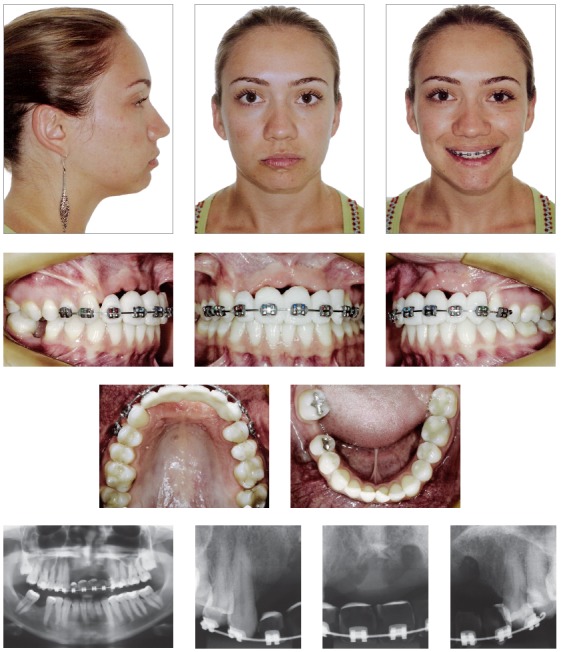
Pretreatment photographs: intraoral view of the Class II dental relationship; intraoral view of the anterior temporary prostheses; occlusal view of the upper and lower arches. Pretreatment panoramic radiograph confirming the absence of the maxillary incisors, left canine and mandibular right first molar, and the extent of anterior alveolar bone loss. Note the short gingival exposure while smiling and slight asymmetry of the lower lip due to a scar.

**Figure 2:**
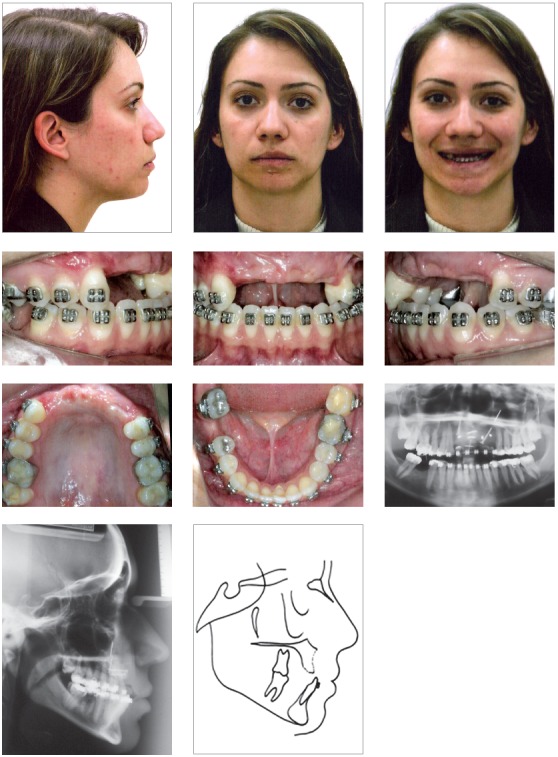
Intraoral photographs following placement of the alveolar bone graft. Panoramic radiograph showing the positioning of the alveolar bone graft.

Clinical examination revealed no evident skeletal disharmony; she had an Angle’s Class II subdivision with crowded mandibular incisors. There was slight asymmetry of her lower lip due to a scar caused by the accident. All permanent maxillary incisors, the left canine, and the mandibular right first molar were absent. Initial panoramic radiograph confirmed the absence of teeth and the extent of alveolar bone loss (Fig 1). The patient had been orthodontically treated, and an alveolar bone graft from the patient’s iliac crest had already been placed. One year after this treatment, she was not satisfied with the treatment outcomes and sought a second treatment option (Fig 2). 

### Treatment objectives

The treatment objectives were to: 1) open enough space for mandibular right first molar rehabilitation; 2) align and upright the mandibular incisors; 3) obtain a canine Class I relationship on the right side; 3) establish functional occlusion; and 4) improve the balance of the subnasal region while smiling and, consequently, improve the patient’s self-esteem. Uprighting of the mandibular incisors was necessary to establish the correct positioning of the maxillary implants in the anterior area.

### Treatment alternatives

The patient had three treatment alternatives for loss of the maxillary incisors and bone: 1) alveolar bone grafting, orthodontic treatment and implant rehabilitation; 2) orthodontic treatment and placement of a prosthetic bridge; 3) orthodontic treatment and placement of removable partial dentures. It is important to consider the biological and functional factors, aesthetics, and costs when determining the best treatment option. 

The steps, benefits and risks of the procedure were explained to the patient, and written informed consent was obtained prior to treatment. After consent was obtained, a multidisciplinary approach involving the previous alveolar bone grafting, orthodontic treatment, and placement of five dental implants was selected for the maxilla, and placement of one dental implant was planned for the mandible. The main risks in this case involved the success of bone grafting and osseointegration of the dental implants. The major treatment challenge was achievement of satisfactory gingival aesthetics because of the extent of the edentulous area. The minimal gingival exposure during smiling was an advantage.

### Treatment progress

**Figure 3:**
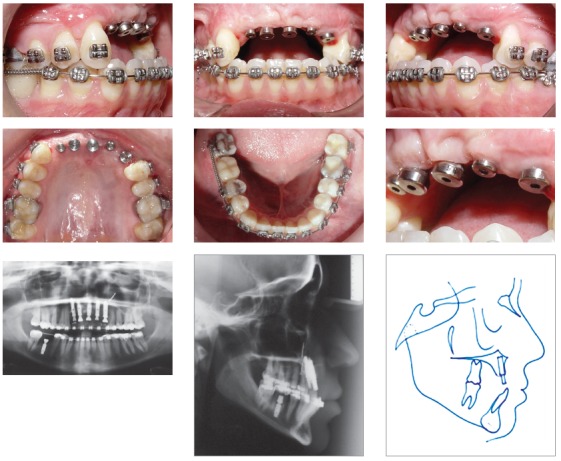
Intraoral photographs showing the replaced mandibular right first molar and the Class I canine relationship. Frontal and lateral views of the five dental implants placed in the anterior area. Final panoramic radiograph showing the dental implants.

**Figure 4:**
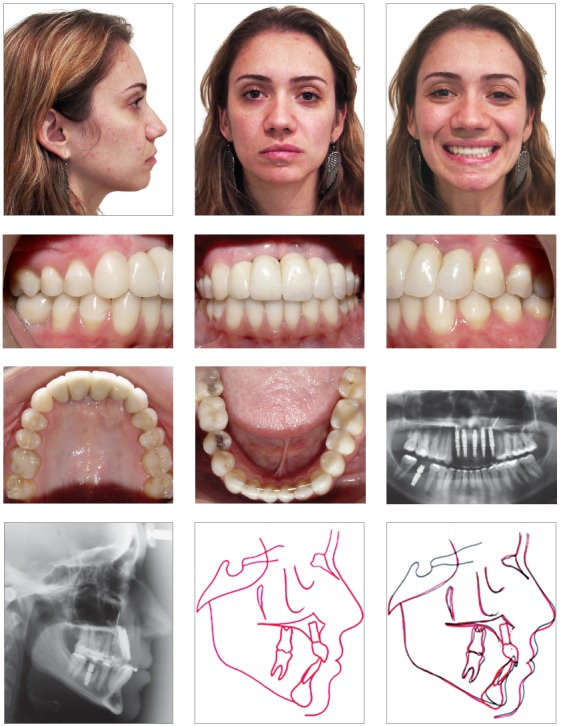
Final photographs, radiographies and superimposition: the prosthesis were placed, and aesthetic and functional rehabilitation was achieved.

A 0.022-in standard non-torqued, non-angulated bonded Edgewise appliance was used. Alignment and leveling of the maxillary teeth were performed, as well as placement of a removable appliance in the anterior region, to maintain the space and improve aesthetics. Posteriorly, artificial teeth were bonded to the brackets and fixed on a 0.019 x 0.025-in rectangular archwire to function as temporary prostheses. In the mandibular arch, the crowded incisors were aligned and leveled. An open coil spring was used to open the space for the dental implant by moving the right first molar to the distal aspect and moving the canines to the mesial aspect, to obtain a Class I canine relationship (Fig 3). Improvement in mandibular incisor positioning was necessary to establish the correct position of the maxillary implants in the anterior area. Finally, the prosthesis was created with a more convex shape in the cervical region of the maxillary incisors, to support the subnasal region, and aesthetic and functional rehabilitation was completed (Fig 4).

### Treatment results

**Figure 5:**
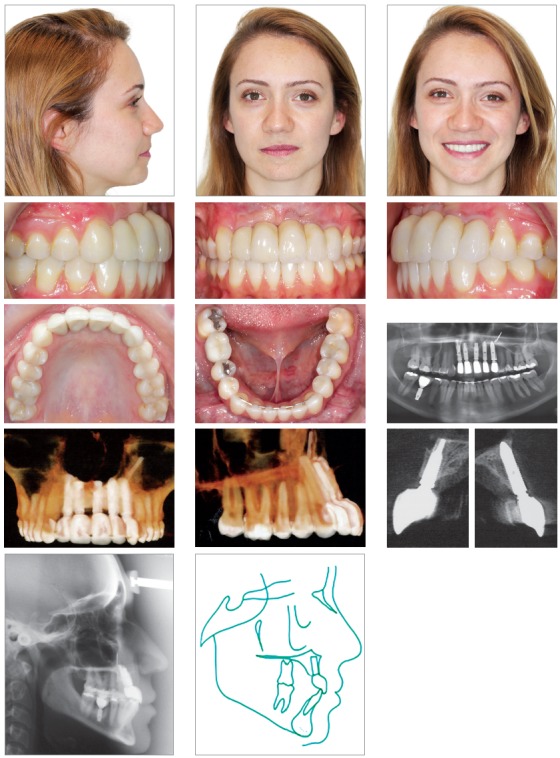
Two-year follow-up photographs demonstrating the maintenance of occlusal stability. CBCT with the implant position.

Once the correct occlusion was established, five dental implants were placed in the anterior area, and one was placed in the right first molar area. The prostheses were placed to complete the aesthetic and functional rehabilitation (Fig 4). The long-term maintenance of these results after 2 years of follow-up (Table 1), with a pleasing smile and adequate overbite and overjet, and adequate positioning of the implants are shown in Figure 5.

**Table 1:** Cephalometric measurements.

Measurements	Pretreat	Pretreat	Posttreat	Follow-up
SNA (degrees)	76	77	78	77
SNB (degrees)	75	75	74	75
ANB (degrees)	1	2	4	2
Ao-Bo (mm)	-1	-1	-1	-1
Facial angle (degrees)	85	86	85	86
Convexity (degrees)	0	2	4	3
FMA (degrees)	25	28	28	25
GoGn-SN) (degrees)	36	38	38	37
Y-Axis (degrees)	59	59	61	60
1-NA (mm)	-	-	7	10
1.NA (degrees)	-	13	16	16
1-NB (mm)	7	-	7	7
1.NB (degrees)	24	27	27	29
Interincisal angle (degrees)	-	138	135	128
Z-angle (degrees)	67	67	70	67

## DISCUSSION

The main goals in this case report were to show the residual defects associated with the loss of anterior teeth and alveolar bone and the subsequent successful placement of implants at those sites. A complicating factor in such cases is the need to maintain functional and aesthetic harmony with adjacent natural teeth.^9^ This report demonstrates successful orthodontic treatment using a multidisciplinary approach in an adult patient with the loss of five anterior teeth due to a car accident. 

Maxillofacial and dental injuries are not uncommon. The epidemiology of maxillofacial trauma includes variable types, severities, and causes depending on the population studied.^10^ ^,^ ^11^ Fractures and injuries of the facial skeleton, as well as anterior tooth loss, are common components of multiple traumas resulting from motor vehicle accidents.^10^ ^,^ ^12^ ^,^ ^13^ Treatment of a traumatized tooth requires fastidious diagnosis and coordination among all treating dental professionals from the moment of injury.

Severe dentoalveolar trauma is often associated with tooth loss, root resorption, and defects in the alveolar crest.^14^ A reduction in alveolar bone mass may have a considerable impact on future treatment options. Implants, resin-bonded bridges, and dentures all require adequate bone mass for the successful and aesthetic replacement of missing anterior teeth.^15^

Treatment planning in reconstructive surgery is crucial. Clinical examination of the traumatized area and soft tissue is also important for choosing the right therapy. In the present case, the patient had lost a large amount of bone mass in the maxillary anterior area. Because of its osteogenic properties and compatibility, autogenous bone grafting is considered the ‘gold standard’ for patients requiring this type of reconstructive surgery.^16^ Because rehabilitation with implants was therefore possible, autogenous bone graft taken from the iliac crest was successfully performed in the present case report. 

Once a list of problems is generated, treatment goals must be established by all professionals involved in the case. In defining the treatment goals, the limitations of the specific case should be taken into consideration to avoid unrealistic expectations.^17^ Due to the severe trauma, the patient in the present case had substantial soft and hard tissues deficiency, and she was appropriately advised regarding the aesthetic limitations of her treatment. Many patients may benefit from orthodontic treatment before implantation and prosthetic restoration and thus achieve more ideal aesthetic and functional results.^18^ When the mandibular incisors were correctly positioned and the space for the right first molar was recovered, the patient was referred for implant placement. She remained with the orthodontic appliances and provisional teeth for 4 months to allow the titanium implants to osseointegrate. After the implants were uncovered and tested for stability, the orthodontic appliances were removed, and temporary crowns were placed on the implants by the restorative dentist. 

The main aesthetic objectives of implant therapy from a surgical point of view are the achievement of a harmonious gingival margin without abrupt changes in tissue height, maintenance of intact papillae, and acquisition or preservation of a convex contour of the alveolar crest.^5^ ^,^ ^19^ Spear and Kokich^20^ described a similar case involving restoration with implants in the lateral incisors region and a four-unit implant-supported bridge. Aspects related to restoration - including midline deviation, buccal-lingual inclination of the maxillary incisors, incisal plane discrepancy, and the smile arch - must also be taken into consideration,^20^ as illustrated in this case report.

The three most significant negative long-term aesthetic outcomes after the replacement of missing incisors by implants are as follows: 1) darkening (blue coloring) of the overlying labial gingival tissue; 2) progressive infraocclusion of the crown (even in older adults); and 3) gingival recession and root exposure.^21^ Aesthetic factors involving gingival tissue are increasingly detrimental to the extent that the patient shows soft tissue when smiling. In this case report, the patient’s minimal gingival smile was an advantageous point for both our immediate approach and long-term aesthetic results. Progressive infraocclusion will probably not be a problem in this case because the four anterior teeth were replaced.

In establishing a diagnosis and treatment plan, well-defined aesthetic objectives must be considered, in addition to impacts on function, structure, and biology. The clinician can use the various specialties of dentistry to achieve the best results for each patient, as shown in this case report.

To establish desirable occlusion and intercuspation with proper inclination of the teeth in this patient, orthodontic therapy alone was ineffective, and a multidisciplinary approach was required. For this patient, bone grafting from the iliac crest was performed to increase the bone height. Five dental implants were placed in the anterior area, and from an aesthetic point of view, the prosthesis was made with a more convex shape at the surface of the maxillary incisors to support the subnasal region. Aesthetic and functional rehabilitation with canine-guided occlusion was achieved, and the patient was pleased with the results.

## CONCLUSION

Anterior aesthetic and functional rehabilitation was successfully achieved in this adult patient with severe dental trauma, including bone loss and the loss of five anterior teeth, due to a car accident. A multidisciplinary approach was essential to improve the patient’s facial aesthetics, to obtain great improvements in function and to achieve occlusal stability after two years of follow-up.
